# 3-hydroxy-3-methylglutaryl-coenzyme A lyase deficiency: one disease - many faces

**DOI:** 10.1186/s13023-020-1319-7

**Published:** 2020-02-14

**Authors:** Sarah C. Grünert, Jörn Oliver Sass

**Affiliations:** 1 Department of General Pediatrics, Adolescent Medicine and Neonatology, Medical Center – University of Freiburg, Faculty of Medicine, Mathildenstr. 1, 79106 Freiburg, Germany; 2 Research Group Inborn Errors of Metabolism, Department of Natural Sciences & Institute for Functional Gene Analytics (IFGA), Bonn-Rhein-Sieg University of Applied Sciences, von-Liebig-Str. 20, 53359 Rheinbach, Germany

**Keywords:** Ketogenesis, Organic aciduria, Leucine, Ketone body, Metabolic acidosis, Hyperammonemia, Hypoglycemia, Metabolic decompensation, HMGCL, Inborn error of metabolism

## Abstract

**Background:**

3-hydroxy-3-methylglutaryl-coenzyme A lyase deficiency (HMGCLD) is an autosomal recessive disorder of ketogenesis and leucine degradation due to mutations in *HMGCL*.

**Method:**

We performed a systematic literature search to identify all published cases. Two hundred eleven patients of whom relevant clinical data were available were included in this analysis. Clinical course, biochemical findings and mutation data are highlighted and discussed. An overview on all published *HMGCL* variants is provided.

**Results:**

More than 95% of patients presented with acute metabolic decompensation. Most patients manifested within the first year of life, 42.4% already neonatally. Very few individuals remained asymptomatic. The neurologic long-term outcome was favorable with 62.6% of patients showing normal development.

**Conclusion:**

This comprehensive data analysis provides a systematic overview on all published cases with HMGCLD including a list of all known *HMGCL* mutations.

## Background

The mitochondrial enzyme 3-hydroxy-3-methylglutaryl-coenzyme A lyase (HMGCL; EC 4.1.3.4) is required not only for the catabolism of the essential branched-chain amino acid leucine, but also for the synthesis of the ketone bodies acetoacetate and 3-hydroxy-*n*-butyrate [1]. Ketone bodies are an important source of energy for extrahepatic organs, in particular of the brain, in times of insufficient energy supply. Consequently, episodes of hypoglycemia and metabolic acidosis are an important observation in HMGCL deficiency (HMGLD; MIM246450). Due to the accumulation of characteristic leucine metabolites HMGCLD can be diagnosed via urinary organic acid analysis and usually is associated with an abnormal blood acylcarnitine profile as well. Confirmatory testing is available by enzyme activity assays in patient cells and by mutation analysis of the *HMGCL* gene. Recently, the number of individuals with confirmed HMGCLD has been estimated to be approximately 200 world-wide [1], but most information published so far is from case reports and small retrospective case series. Few studies have presented larger patient cohorts [2–8]. Although many patients reported originate from the Iberic peninsula and from Saudi Arabia, where HMGCLD is the most prevalent organic aciduria, HMGCLD is a panethnic disease. However, a comprehensive meta-analysis that covers all HMGCLD patients described in the literature so far is missing.

This has prompted us to approach a systematic assessment of all described patients with this ketogenesis defect.

## Methods

We performed a systematic literature search in PubMed using the terms “3-Hydroxy-3-methylglutaryl-coenzyme A lyase deficiency”, “3-Hydroxy-3-methylglutaryl-coA lyase deficiency”, “HMGCL deficiency”, and “3-HMG-coenzyme A lyase deficiency” in order to obtain information on the clinical course of all published patients. The search was performed in September 2019 and was completed by searches in the Human Gene Mutation Database (HGMD®) http://www.hgmd.cf.ac.uk/. This was supplemented by patient data from literature known to the authors due to their long-time work in the area of ketone body metabolism. All patients with metabolically, enzymatically and/or genetically proven HMGCLD on whom relevant clinical information was provided in the respective publication(s) were included in this study. With this approach we identified a total of 211 HMGCLD patients, mainly published in case reports as well as few case series. All cases were evaluated and analysed with a special focus on the patients’ age at onset, number of metabolic decompensations, clinical course including neurological outcome, treatment, residual enzyme activity and mutations in the *HMGCL* gene.

For the systematic overview on all published *HMGCL* mutations, publications that contained no clinical information were included in addition. A list of all publications included in the clinical data analysis is given in Additional file 1: Table S1, a list of publications that were additionally reviewed for mutations is provided in Additional file 2: Table S2.

Accuracy of age data ranged from hours to years in the different reports. For the calculation of median ages years were converted to months which might lead to an underestimation (i.e. 7 years = 84 months, although the patient might have been 7 years and 11 months old). In very few cases where only “newborn” was given for the age at report, we used 5 days of age for the calculation. If hours were given (“first symptoms 2 hours after birth”) these were rounded to days.

## Results

Two hundred eleven cases of HMGCLD were identified and reviewed (Table 1, Additional file 1: Table S1). Seventy-eight patients were female, 101 were male, and the sex of the remaining 32 patients was not reported. The dataset included 8 pairs of siblings, thereof one pair of dizygotic twins. The age at last reported clinical follow-up was provided for 155 patients and ranged from 72 h to 40 years (median of 48 months). Forty-six patients were of Turkish origin, 20 Portuguese, 13 Brazilian of Portuguese ancestry, 19 Saudi Arabian, and 12 Spanish. All other origins accounted for 7 or less patients, and of 20 patients no ethnic or geographic origin was reported. Information on parental consanguinity was given in 122 cases with a consanguinity rate of 49% (60/122). 169 (80.1%) patients were alive at the time of report, 34 (16.1%) patients had deceased, and of 8 patients the outcome is not reported. Age at death ranged from 72 h to 40 years (median 9.5 months, *n* = 26). Most patients died due to metabolic decompensations, one of them at the age of 24 years during her second pregnancy [9]. One child deceased in his sleep at age 13 months with no apparent previous symptoms. One previously asymptomatic 29-year-old patient died of septic shock with multi-organic failure [10], one 7 month-old child due to cardiomyopathy and arrhythmias [11].

**Table 1:** Clinical information on 211 published patients with HMGCL deficiency

Sex	female *n* = 78 male *n* = 101 not reported *n* = 32
Age at last clinical follow-up	median 48 months (*n* = 155, range: 72 h to 40 years)
Parental consanguinity	49% (60/122)
Deceased patients	16.1% (34/211)
Median age at disease onset	4 months (*n* = 146)
Patients with at least 1 metabolic decompensation	95.3% (163/171)
Patients with normal psychomotor development	62.6% (87/139)
Patients with developmental delay or distinct neurologic abnormalities	31.7% (44/139)

Information on the number of metabolic decompensations was available for 171 patients. Thereof, 163 patients (95.3%) suffered at least one metabolic crisis. In 8 patients more than 10 acute episodes were reported. Eight patients never had metabolic decompensations. Two of these patients were diagnosed asymptomatically by family screening [12, 13], three presented with seizures and/or developmental delay [2, 14–16] and two were diagnosed due to hepatomegaly and elevated plasma/serum activities of transaminases [2, 5]. One patient presented with macrocephaly, which was first noted at 2 months, as well as a doll-like facies with frontal bossing and depressed nasal bridge [17]. She also displayed a slight “sun-setting” phenomenon, tendency to opisthotonus and global developmental delay. Although this child never had a metabolic crisis a tendency towards hypoglycemia was reported [17–20]. One patient was diagnosed presymptomatically through family screening, but developed an acute decompensation in the third year of life [12].

**Fig. 1:**
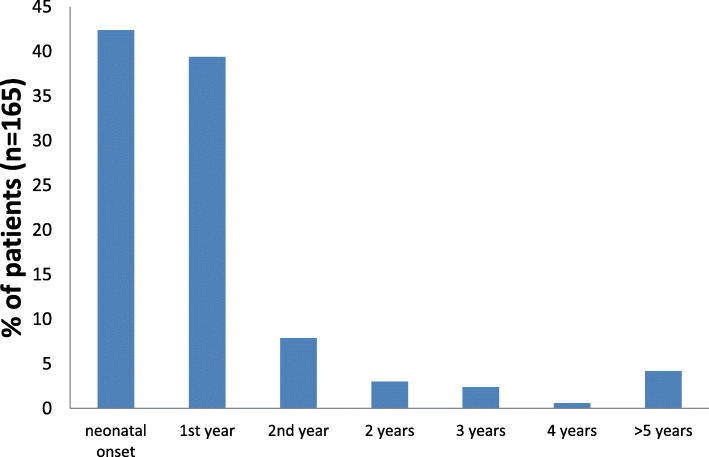
Age at first presentation of 165 HMGCLD patients with acute symptoms. The vast majority of patients presented within the first year of life with neonatal onset in more than 40% of patients. The latest manifestation was observed at 29 years

Of 165 patients with acute symptoms the age at presentation was reported (146 cases with exact numbers, and 20 with some information, such as “neonatal onset” or “presentation in the third year of life). Median age at disease onset was 4 months (*n* = 146). 70/165 (42.4%) patients presented neonatally, 65 (39.4%) and 13 (7.9%) in the first and second year of life, respectively, while the remainder of 17 patients (17/165; 10.8%) showed first symptoms beyond the second year of life only (Fig. 1). Within the neonatal onset group 11 patients were already symptomatic on the first day of life. The latest manifestation was reported in a 29-year-old patient who died during the initial metabolic crisis due to multiorgan failure [10]. There was often a significant delay until the correct diagnosis could be made. In one patient it took 36 years between the onset of symptoms and the time of diagnosis [2, 21].

Clinical symptoms of acute decompensations mainly comprised vomiting, lethargy/ coma, tachypnea/apnoea, seizures and moderate hepatomegaly. Few patients presented with stroke-like episodes. Common laboratory findings were (severe) hypoglycemia, metabolic acidosis, elevated activities of serum transaminases and hyperammonemia. Transaminase activities were often only mildly increased, but episodes of transient elevations up to > 1000 U/l have been reported in few patients [22, 23]. One child developed an episode of liver dysfunction with massively elevated transaminase activities (AST 4150 IU/L, ALT 2200 IU/L) at age 5 months [22], and another patient showed an AST activity of 11,736 IU/l during a severe metabolic decompensation at age 7 months [23]. Hyperammonemia was rather mild in most cases, however ammonia levels > 1000 μmol/L have been described, and one patient even presented with severe hyperammonemia of > 2000 μmol/l requiring peritoneal dialysis [24].

**Fig. 2:**
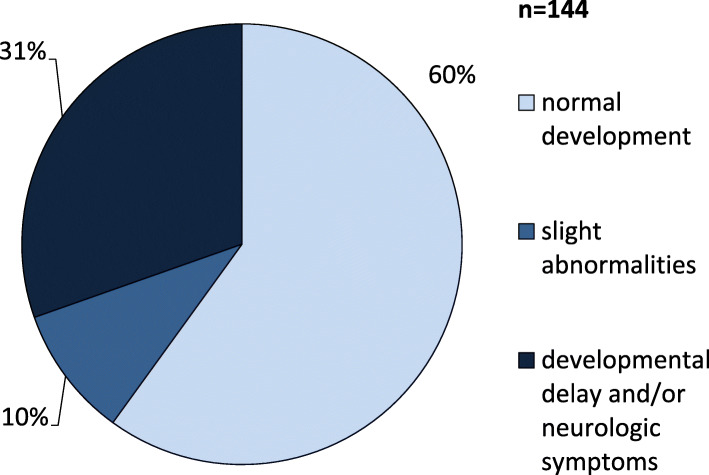
Cognitive development and neurologic complications in 139 HMGCLD patients. 62.6% of patients show normal development, while severe mental disability is rather rare in this patient cohort. Neurologic symptoms were documented in 10 patients including spastic hemiparesis or tetraplegia, distinct muscular hypotonia, impairment of vision and hearing, cerebellar ataxia, movement disorders, tremor, clonic movements, mild dysarthria, exaggerated deep tendon reflexes and absence of social contact. Seizures were reported in 13 patients

Information on the neurologic outcome was available on 140 patients (Fig. 2). Thereof, 87 (87/139; 62.6%) showed normal psychomotor development without neurologic abnormalities. One 2-year-old patient had trisomy 21 [25] and was therefore not included in the analysis. In 9 patients (9/139; 6.5%) only slight abnormalities were reported including muscular hypotonia or a transiently increased muscle tone, hyperactivity and partial performance weaknesses such as dyslexia and difficulties in grammar. Forty-four patients (44/139; 31.7%) showed developmental delay or distinct neurologic abnormalities. Eighteen patients were described as severely retarded, 6 had a moderate and 4 a mild disability. In 5 cases the degree of neurologic impairment was not further specified. Ten patients showed neurologic symptoms including spastic hemiparesis or tetraplegia, distinct muscular hypotonia, impairment of vision and hearing, cerebellar ataxia, movement disorders, tremor, clonic movements, mild dysarthria, exaggerated deep tendon reflexes and absence of social contact. Seizures were reported in 13 patients (9.0%).

Imaging data (MRI or CT) were available of 60 patients. There will of course be a bias in favour of abnormal findings as imaging is primarily performed in patients with neurologic symptoms. Nevertheless, it is notable that imaging results were unremarkable in only 2 children [2, 26]. The most common findings were white matter changes which were uniformly present in almost all patients. Another frequent observation was cerebral atrophy with dilatation of the ventricular system. Abnormalities reported in a few or single patients comprised basal ganglia involvement, demyelinisation, ischemic lesions, chronic subdural hematoma, subdural hygroma and bilateral occipital porencephaly.

In 117 cases information on dietary regimens is available. Nine of these patients (7.7%) had no dietary restrictions, although in one of them a low-leucine diet was recommended. The remaining 108 patients followed a specific diet at least temporarily. Forty-six patients (46/105; 43.8%) were on either a low-leucine (28 patients) or low-protein (18 patients) diet, 57 patients (57/105; 54.3%) followed a diet low in leucine/protein and fat. Only one patient had a fat-restricted diet without protein restriction (1/105; 1%). Few patients had a self-imposed diet already before diagnosis [13, 27–29]. In three cases it was only stated that a diet was given, but no details were provided. Many patients on leucine/ protein restriction received supplementation with a leucine-free amino acid mixture. Some patients received additional carbohydrate supplementation either by corn starch or by glucose polymers. Avoidance of fasting was usually recommended. One patient received long-term treatment with diazoxide (25 mg/8 h) [20]. Some patients were supplemented with bicarbonate. In some patients the diet was relaxed at some point during childhood. For 109 patients, data on carnitine treatment was available. In this cohort, carnitine was supplemented in 85 cases (78%), while 24 (22%) patients received no carnitine supplementation.

Apart from neurologic symptoms long-term complications affecting other organs seem to be rather rare. Three patients developed dilated cardiomyopathy with arrhythmias which were fatal in two cases [2, 11, 30], and in one patient left ventricular noncompaction was diagnosed [31]. Two patients were reported with pancreatitis, one 5-year-old girl with a single episode [31] and one boy with recurrent episodes [22].

In 4 of 216 cases, HMCGL deficiency was reported in association with another congenital disorder. One patient had a trisomy 21 [25], one patient was reported with VATERL syndrome [32], and one patient had a situs inversus totalis and gastroschisis [33]. In the fourth patient who presented with deafness and retinitis pigmentosa an Usher syndrome type I, a rare autosomal recessive condition of profound congenital deafness and severe retinitis pigmentosa associated with developmental delay, was suspected [27].

A total of 8 pregnancies have been reported in 5 women [9, 34–36]. Five pregnancies resulted in healthy offspring, while one mother who already presented with recurrent metabolic decompensations during her first pregnancy died during her second pregnancy at 9 weeks of gestation due to maternal metabolic decompensation [9]. In one patient intrauterine death occurred in the first pregnancy at 10 weeks of gestation during maternal metabolic decompensation, and the second pregnancy was terminated at 6 weeks of gestation in absence of metabolic problems [9].

Enzymatic studies have been performed in 114 patients confirming a reduced or absent HMGCL activity in leukocytes, Epstein-Barr virus (EBV)-transformed lymphoblastoid cells or fibroblasts in all of them.

**Fig. 3:**
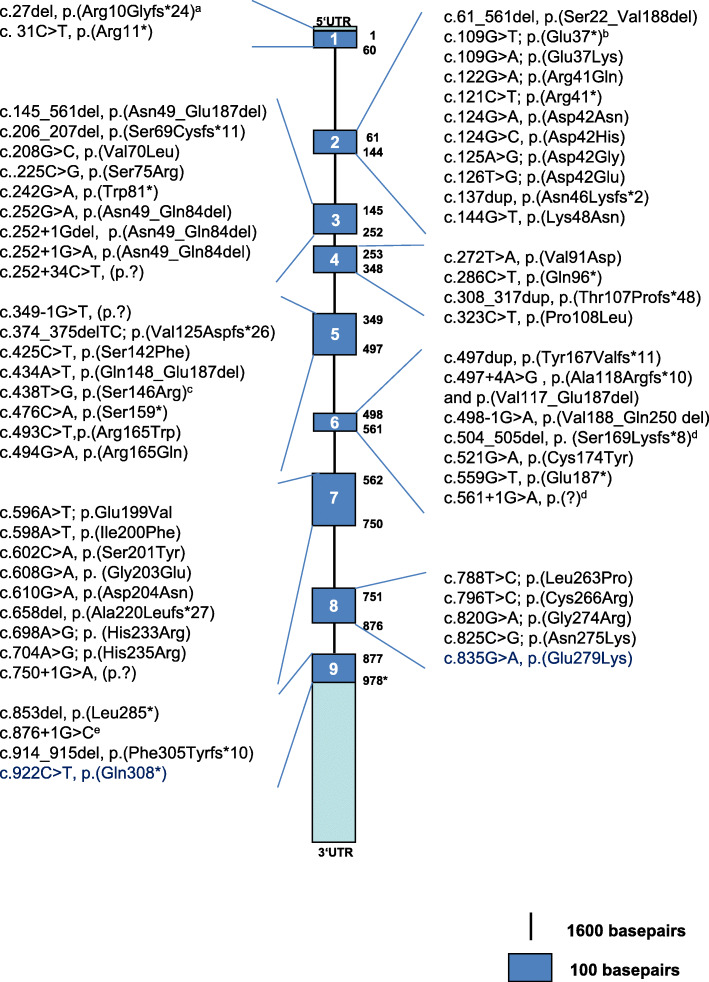
Mutations reported for human *HMGCL*. ^a^: c.27del has been demonstrated by Pospísilová et al. 2003 to lead to a frame shift [39]. This mutation leads to a frameshift and premature stop codon after 32 amino acids without degradation of the DNA, while p.(Arg10Glyfs*24) would be predicted. ^b^: associated with skipping of exon 2 [15]. ^c^: Likely to affect splicing, although not proven [40]. ^d^: Has been named p.Val168Valfs8 by Puisac et al. 2013; may also cause skipping of exon 5 or of exons 5 and 6 (the latter resulting in a physiological mRNA transcript according to [41]. ^e^: According to Buesa et al. 1996 aberrant splicing, which mostly results in skipping of exon 9, p.(Met251_Thr292del,), but to a small extent in insertion of 17 amino acids, which precede a stop codon: p.(fs*18) [42]. Not displayed: -Pie et al. (1997) reported a 84 bp in-frame deletion on the mRNA level leads to the loss of 28 amino acids (Val-21 to Lys-48) in the mature protein [43]. This deleted region includes the last of the leader peptide of the precursor HL protein and 21 amino acids of the N-terminus of the mature protein. -Deletion (between intron 1 and intron 4) NG_013061: g.9326_13806del reported by Aoyama et al. 2015 [44]. - Mutation r.61-144del identified on the RNA level only [2]. - As the skipping of exons 5-6b skipping of exons 5–7 has been reported for a physiological alternative transcript [41]. Note: Zaferiou et al. 2007 referred to a ‘C-to-T transition’ which actually should be c.796C > T and is indicated as such in this figure [45]. Roland et al. 2017: c.438 T > G, p.(Ser46Arg) was corrected to p.(Ser146Arg) [37]

Results of *HMGCL* mutation analysis were reported for 118 patients. Mutations were identified in all 9 exons of *HMGCL* and also in noncoding regions of the gene. An overview on all mutations identified in the *HMCGL* gene that were reported in the literature so far is given in Fig. 3 (following transcription into the current nomenclature, where required). Eighty-six patients (72.9%) carried homozygous mutations and 24 patients (20.3%) were compound heterozygous for variants in the *HMGCL* gene. In one of the homozygous patients, paternal uniparental isodisomy of chromosome 1 was confirmed (Aoyama 2015). In 6 patients (5.1%) only one mutation was detected, and in 2 patients (1.7%) no mutation could be identified although HMGCL activity was clearly deficient in fibroblasts [2, 37]. The most common *HMGCL* mutation was the c.109G > T, p.(Glu37*) variant that was found in a total of 36 patients (30.5%), mostly in homozygosity (homozygous in 28 patients, heterozygous in 8 patients). It was mostly reported in individuals originating from the geographically/ demographically linked countries Brazil, Spain, Portugal and Morocco, but also in two Pakistani patients. The two other common variants that were identified in 12 (10.2%) and 6 patients (5.5%), respectively, were c.122G > A, p.(Arg41Gln) and the splice site mutation c.876 + 1G > C. While p.(Arg41Gln) was mostly found in individuals from Saudi Arabia, it was also noted in individuals of Turkish and Italian origin. c.876 + 1G > C is common among Turkish patients with HMGCLD. All other mutations were reported in less than 5 individuals.

## Discussion/conclusion

This work aims at a comprehensive overview on the clinical course, biochemical and genetic data of all patients with HMGCLD published in the literature so far.

Patients with HMGCLD typically present with acute metabolic decompensation that may be life-threatening. Very few patients were diagnosed with only chronic, mainly neurologic symptoms. Interestingly, only very few asymptomatic patients have been described although HMGCLD is a target disease of newborn screening programs in several countries. This may of course be due to a publication bias as asymptomatic individuals are often not reported, and some of the individuals identified by newborn screening were described in papers with no further relevant clinical information and therefore not included in this analysis.

Of the symptomatic patients 42.4% presented neonatally and more than 80% within the first year of life, while manifestation beyond the first year of life was the exception. This is compatible with the special role of ketone bodies for the energy supply of the newborn. Despite the often early and severe manifestation the long-term outcome seems to be favourable with the majority of patients showing normal cognitive development. Taking into consideration that our analysis also included patients that were diagnosed and treated as early as in the 1970s, the prognosis of patients born today may be even better than assumed based on this cohort.

As in other rare inherited metabolic diseases no controlled treatment studies are available for HMCGLD. Therefore, no conclusions can be drawn with respect to the necessity of a special diet or carnitine supplementation from our data although the majority of patients was on a protein and/or fat restricted diet. Based on pathobiochemical considerations and clinical reports the avoidance of fasting seems to be the mainstay of therapy in this disorder of ketogenesis. Administration of L-carnitine may have detoxifying effects and help to avoid secondary L-carnitine deficiency and intracellular depletion of free coenzyme A [38].

Our data demonstrate that HMGCLD is a panethnic disease, although some mutations are clustered in certain geographic areas with close connections throughout history. Interestingly, 18 patients carrying a homozygous mutation in *HMGCL* were explicitly reported to be the offspring of a non-consanguineous union. This possibly reflects an underestimation of parental consanguinity in this patient cohort. In line with previous reports on patient subgroups, our comprehensive study underlines that “genotype–phenotype correlations are difficult to establish” in HMGCLD [2, 6].

## Conclusion

Despite its often early and severe manifestation HMGCLD seems to be associated with a favorable long-term outcome in the majority of cases.

## Supplementary information

**Additional file 1: Table S1.** Publications included in this literature review for the analysis of clinical, biochemical and genetic data.

**Additional file 2: Table S2.** Publications that were additionally included for the overview on all known mutations in *HMGCL.*

## Data Availability

Raw data of this analysis are available on request.

## Abbreviations

HMGCL: 3-hydroxy-3-methylglutaryl coenzyme A lyase
HMGCLD: 3-hydroxy-3-methylglutaryl coenzyme A lyase deficiency
