# Correction to: 3-hydroxy-3-methylglutaryl-coenzyme A lyase deficiency: one disease - many faces

**DOI:** 10.1186/s13023-021-02154-z

**Published:** 2022-01-10

**Authors:** Sarah C. Grünert, Jörn Oliver Sass

**Affiliations:** 1 Department of General Pediatrics, Adolescent Medicine and Neonatology, Medical Center – University of Freiburg, Faculty of Medicine, Mathildenstr. 1, 79106 Freiburg, Germany; 2 Research Group Inborn Errors of Metabolism, Department of Natural Sciences & Institute for Functional Gene Analytics (IFGA), Bonn-Rhein-Sieg University of Applied Sciences, von-Liebig-Str. 20, 53359 Rheinbach, Germany

## Correction to: Orphanet Journal of Rare Diseases (2020) 15:48 10.1186/s13023-020-1319-7

In the original article [1], data labelling in Fig. 2 was incorrect by mistake. The total number of patients with data on neurological outcome available was n = 139. The corrected figure is shown below.

The figure legend should read:

**Fig. 2:**
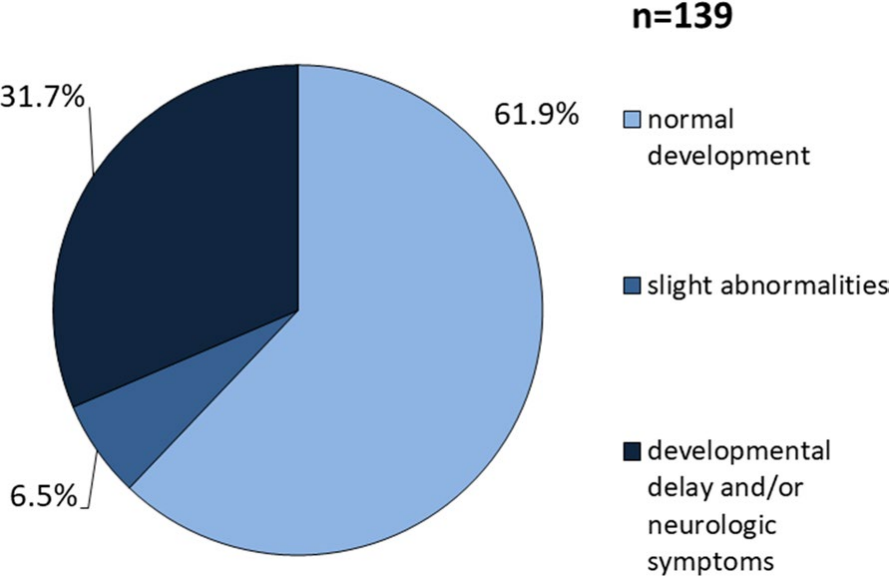
Cognitive development and neurologic complications in 139 HMGCLD patients. 61.9% of patients show normal development, while severe mental disability is rather rare in this patient cohort. Neurologic symptoms were documented in 10 patients including spastic hemiparesis or tetraplegia, distinct muscular hypotonia, impairment of vision and hearing, cerebellar ataxia, movement disorders, tremor, clonic movements, mild dysarthria, exaggerated deep tendon reflexes and absence of social contact. Seizures were reported in 13 patients

The paragraph on the neurological outcome on page 3 also contains one wrong number and should read “Information on the neurologic outcome was available on 140 patients (Fig. 2). One 2-year-old patient had trisomy 21 [25] and was therefore not included in the analysis. 86 (86/139; 61.9%) showed normal psychomotor development without neurologic abnormalities. In 9 patients (9/139; 6.5%) only slight abnormalities were reported…”

The authors apologize for these errors and state that these do not change the scientific conclusions of the article in any way.
