# Supplementary material for: 3-hydroxy-3-methylglutaryl-coenzyme A lyase deficiency: one disease - many faces
Source: Orphanet J Rare Dis. 2020 Feb 14;15:48. doi: 10.1186/s13023-020-1319-7 (PMC7023732; doi:10.1186/s13023-020-1319-7)
Supplement: Supplementary file 1 — Additional file 1: Table S1. Publications included in this literature review for the analysis of clinical, biochemical and genetic data. [file 13023_2020_1319_MOESM1_ESM.docx]

**Table S1: Publications included in this literature review for the analysis of clinical, biochemical and genetic data.**

1. al-Essa M, Rashed M, Ozand PT. 3-Hydroxy-3-methylglutaryl-CoA lyase deficiency in a boy with VATER association. J Inherit Metab Dis. 1998;21:443–4.

2. Alharby, E, Malibari, O, Al-Otaibi, HS, Saud, M, Al-Harbi, G, Samman, MI, et al. Recurrent mutation in the HMGCL gene in a family segregating HMG-CoA lyase deficiency. International Journal of Genetics and Molecular Biology. 2016;8:11–7.

3. Aoyama Y, Yamamoto T, Sakaguchi N, Ishige M, Tanaka T, Ichihara T, et al. Application of multiplex ligation-dependent probe amplification, and identification of a heterozygous Alu-associated deletion and a uniparental disomy of chromosome 1 in two patients with 3-hydroxy-3-methylglutaryl-CoA lyase deficiency. Int J Mol Med. 2015;35:1554–60.

4. Applegarth DA, MacLeod PM, Toone JR, Kirby LT, MacLean JR, Mamer OA, et al. Organic acids and Reye’s syndrome. Lancet. 1979;1:1147.

5. Bakker HD, Wanders RJ, Schutgens RB, Abeling NG, van Gennip AH. 3-Hydroxy-3-methylglutaryl-CoA lyase deficiency: absence of clinical symptoms due to a self-imposed dietary fat and protein restriction. J Inherit Metab Dis. 1993;16:1061–2.

6. Barash V, Mandel H, Sella S, Geiger R. 3-Hydroxy-3-methylglutaryl-coenzyme A lyase deficiency: biochemical studies and family investigation of four generations. J Inherit Metab Dis. 1990;13:156–64.

7. Bhattacharya, K, Ho, G, Dalkeith, T, Dennison, B, Thompson, S, Christodoulou, J. Improvement in severe HMG co-lyase deficiency with fat restriction and 3-hydroxybutyrate therapy. J Inherit Metab Dis. 2010;33 (Suppl 1):62.

8. Bischof F, Nägele T, Wanders RJA, Trefz FK, Melms A. 3-hydroxy-3-methylglutaryl-CoA lyase deficiency in an adult with leukoencephalopathy. Ann Neurol. 2004;56:727–30.

9. Buesa C, Pié J, Barceló A, Casals N, Mascaró C, Casale CH, et al. Aberrantly spliced mRNAs of the 3-hydroxy-3-methylglutaryl coenzyme A lyase (HL) gene with a donor splice-site point mutation produce hereditary HL deficiency. J Lipid Res. 1996;37:2420–32.

10. Cardoso ML, Rodrigues MR, Leão E, Martins E, Diogo L, Rodrigues E, et al. The E37X is a common HMGCL mutation in Portuguese patients with 3-hydroxy-3-methylglutaric CoA lyase deficiency. Mol Genet Metab. 2004;82:334–8.

11. Casale CH, Casals N, Pié J, Zapater N, Pérez-Cerdá C, Merinero B, et al. A nonsense mutation in the exon 2 of the 3-hydroxy-3-methylglutaryl coenzyme A lyase (HL) gene producing three mature mRNAs is the main cause of 3-hydroxy-3-methylglutaric aciduria in European Mediterranean patients. Arch Biochem Biophys. 1998;349:129–37.

12. Casals N, Pié J, Casale CH, Zapater N, Ribes A, Castro-Gago M, et al. A two-base deletion in exon 6 of the 3-hydroxy-3-methylglutaryl coenzyme A lyase (HL) gene producing the skipping of exons 5 and 6 determines 3-hydroxy-3-methylglutaric aciduria. J Lipid Res. 1997;38:2303–13.

13. Casals N, Gómez-Puertas P, Pié J, Mir C, Roca R, Puisac B, et al. Structural (betaalpha)8 TIM barrel model of 3-hydroxy-3-methylglutaryl-coenzyme A lyase. J Biol Chem. 2003;278:29016–23.

14. Dasouki M, Buchanan D, Mercer N, Gibson KM, Thoene J. 3-Hydroxy-3-methylglutaric aciduria: response to carnitine therapy and fat and leucine restriction. J Inherit Metab Dis. 1987;10:142–6.

15. Divry, P, Rolland MO, Teyssie, J, Cotte, J. 3-Hydroxy-3-methylglutaric Aciduria Combined with 3-Methylglutaconic Aciduria: A New Case. J Inherit Metab Dis. 1981;4:173–4.

16. Dodelson de Kremer R, Kelley RI, Depetris de Boldini C, Paschini de Capra A, Corbella L, Givogri I, et al. [3-hydroxy-3-methylglutaryl-coenzyme A lyase deficiency as a cause of severe neurological damage]. Medicina (B Aires). 1992;52:30–6.

17. Duran M, Ketting D, Wadman SK, Jakobs C, Schutgens RB, Veder HA. Organic acid excretion in a patient with 3-hydroxy-3-methylglutaryl-CoA lyase deficiency: facts and artefacts. Clin Chim Acta. 1978;90:187–93.

18. Duran M, Schutgens RB, Ketel A, Heymans H, Bertssen MW, Ketting D, et al. 3-hydroxy-3-methylglutaryl coenzyme A lyase deficiency: postnatal management following prenatal diagnosis by analysis of maternal urine. J Pediatr. 1979;95:1004–7.

19. Eirís J, Ribes A, Fernández-Prieto R, Rodríguez-García J, Rodríguez-Segade S, Castro-Gago M. [3-hydroxy-3-methylglutaric aciduria and recurrent Reye-like syndrome]. Rev Neurol. 1998;26:911–4.

20. Faull K, Bolton P, Halpern B, Hammond J, Danks DM, Hähnel R, et al. Letter: Patient with defect in leucine metabolism. N Engl J Med. 1976;294:1013.

21. Ferreira G, Freitas S, Pereira SA, Martins I, Tavares E, Vilarinho L. 3-Hydroxy-3-methylglutaric aciduria in a girl with trisomy 21. Eur J Pediatr. 1996;155:1068.

22. Ferris NJ, Tien RD. Cerebral MRI in 3-hydroxy-3-methylglutaryl-coenzyme A lyase deficiency: case report. Neuroradiology. 1993;35:559–60.

23. Francois, B, Bachmann, C, Schutgens, RBH. Glucose Metabolism in a Child with 3-Hydroxy- 3-Methylglutaryl-Coenzyme A Lyase Deficiency. J Inherit Metab Dis. 1981;4:163–4.

24. Funghini S, Pasquini E, Cappellini M, Donati MA, Morrone A, Fonda C, et al. 3-Hydroxy-3-methylglutaric aciduria in an Italian patient is caused by a new nonsense mutation in the HMGCL gene. Mol Genet Metab. 2001;73:268–75.

25. Gibson KM, Breuer J, Kaiser K, Nyhan WL, McCoy EE, Ferreira P, et al. 3-Hydroxy-3-methylglutaryl-coenzyme A lyase deficiency: report of five new patients. J Inherit Metab Dis. 1988;11:76–87.

26. Gibson KM, Breuer J, Nyhan WL. 3-Hydroxy-3-methylglutaryl-coenzyme A lyase deficiency: review of 18 reported patients. Eur J Pediatr. 1988;148:180–6.

27. Gibson KM, Cassidy SB, Seaver LH, Wanders RJ, Kennaway NG, Mitchell GA, et al. Fatal cardiomyopathy associated with 3-hydroxy-3-methylglutaryl-CoA lyase deficiency. J Inherit Metab Dis. 1994;17:291–4.

28. Gibson KM, Sweetman L, Nyhan WL, Page TM, Greene C, Cann HM. 3-hydroxy-3-methylglutaric aciduria: a new assay of 3-hydroxy-3-methylglutaryl-coa lyase using high performance liquid chromatography. Clin Chim Acta. 1982;126:171–81.

29. Gibson KM, Lee CF, Kamali V, Johnston K, Beaudet AL, Craigen WJ, et al. 3-Hydroxy-3-methylglutaryl-CoA lyase deficiency as detected by radiochemical assay in cell extracts by thin-layer chromatography, and identification of three new cases. Clin Chem. 1990;36:297–303.

30. Gordon K, Riding M, Camfield P, Bawden H, Ludman M, Bagnell P. CT and MR of 3-hydroxy-3-methylglutaryl-coenzyme A lyase deficiency. AJNR Am J Neuroradiol. 1994;15:1474–6.

31. Greene CL, Cann HM, Robinson BH, Gibson KM, Sweetman L, Holm J, et al. 3-Hydroxy-3-methylglutaric aciduria. J Neurogenet. 1984;1:165–73.

32. Grünert SC, Schlatter SM, Schmitt RN, Gemperle-Britschgi C, Mrázová L, Balcı MC, et al. 3-Hydroxy-3-methylglutaryl-coenzyme A lyase deficiency: Clinical presentation and outcome in a series of 37 patients. Mol Genet Metab. 2017;121:206–15.

33. Hammond J, Wilcken B. 3-hydroxy-3-methylglutaric, 3-methylglutaconic and 3-methylglutaric acids can be non-specific indicators of metabolic disease. J Inherit Metab Dis. 1984;7 Suppl 2:117–8.

34. Huemer M, Muehl A, Wandl-Vergesslich K, Strobl W, Wanders RJ, Stoeckler-Ipsiroglu S. Stroke-like encephalopathy in an infant with 3-hydroxy-3-methylglutaryl-coenzyme A lyase deficiency. Eur J Pediatr. 1998;157:743–6.

35. Jones KJ, Wilcken B, Kilham H. The long-term evolution of a case of 3-hydroxy-3-methylglutaryl-coenzyme A lyase deficiency associated with deafness and retinitis pigmentosa. J Inherit Metab Dis. 1997;20:833–4.

36. Karcher C, Rousselot JM, Lefebvre E, Vidailhet M. [Hydroxy-methyl-glutaryl-coenzyme A lyase deficiency manifesting as Reye’s syndrome in a 3-year-old girl]. Pediatrie. 1993;48:385–7.

37. Kasapkara, CS, Akar, M, Biberoglu, G, Celik, M, Özbek, MN, Tüzün, H. When a common symptom of a neonate becomes an unusual diagnosis: A case report of HMG-CoA lyase deficiency. J Inherit Metab Dis. 2014;37 (Suppl 1):180.

38. Ketel A, Ket JL, Schutgens RB, Duran M, Wadman SK. Clinical and biochemical observations on a child with a deficiency of 3-hydroxy-3-methylglutaryl coenzyme A lyase. J Inherit Metab Dis. 1980;3:89–90.

39. Kikuchi M, Narisawa K, Tada K, Sweetman L. Enzymatic diagnosis of 3-hydroxy-3-methylglutaryl-CoA lyase deficiency with high-performance liquid chromatography. Clin Chim Acta. 1990;189:297–301.

40. Köksal T, Gündüz M, Özaydın E, Azak E. 3-HMG Coenzyme A Lyase Deficiency: Macrocephaly and Left Ventricular Noncompaction with a Novel Mutation. Indian J Pediatr. 2015;82:645–8.

41. Koling S, Kalhoff H, Schauerte P, Lehnert W, Diekmann L. [3-hydroxy-3-methylglutaraciduria (case report of a female Turkish sisters with 3-hydroxy-3- methylglutaryl-Coenzyme A lyase deficiency]. Klin Padiatr. 2000;212:113–6.

42. Kose, M, Kagnıcı, M, Canda, E, Altinol, Y, Ceylaner, S, Aalkan, K, et al. HMG-CoA lyase Deficiency: One disease three pictures. J Inherit Metab Dis. 2014; 37 (Suppl 1):S107.

43. Langendonk JG, Roos JCP, Angus L, Williams M, Karstens FPJ, de Klerk JBC, et al. A series of pregnancies in women with inherited metabolic disease. J Inherit Metab Dis. 2012;35:419–24.

44. Lee C, Tsai FJ, Wu JY, Peng CT, Tsai CH, Hwu WL, et al. 3-hydroxy-3-methylglutaric aciduria presenting with Reye like syndrome: report of one case. Acta Paediatr Taiwan. 1999;40:445–7.

45. Leonard JV, Seakins JW, Griffin NK. beta-Hydroxy-beta-methyglutaricaciduria presenting as Reye’s syndrome. Lancet. 1979;1:680.

46. Leung AAC, Chan AK, Ezekowitz JA, Leung AKC. A Case of Dilated Cardiomyopathy Associated with 3-Hydroxy-3-Methylglutaryl-Coenzyme A (HMG CoA) Lyase Deficiency. Case Rep Med. 2009;2009:183125.

47. Leupold D, Bojasch M, Jakobs C. 3-hydroxy-3-methylglutaryl-CoA lyase deficiency in an infant with macrocephaly and mild metabolic acidosis. Eur J Pediatr. 1982;138:73–6.

48. Lin W-D, Wang C-H, Lai C-C, Tsai Y, Wu J-Y, Chen C-P, et al. Molecular analysis of Taiwanese patients with 3-hydroxy-3-methylglutaryl CoA lyase deficiency. Clin Chim Acta. 2009;401:33–6.

49. Lisson G, Leupold D, Bechinger D, Wallesch C. CT findings in a case of deficiency of 3-hydroxy-3-methylglutaryl-CoA-lyase. Neuroradiology. 1981;22:99–101.

50. Ma Y-Y, Song J-Q, Wu T-F, Liu Y-P, Xiao J-X, Jiang Y-W, et al. [Leucodystrophy induced by late onset 3-hydroxy-3-methylglutaric aciduria]. Zhongguo Dang Dai Er Ke Za Zhi. 2011;13:392–5.

51. Marklová E, Verner P, Pehal F, Brátová M, Polák J. A new case of 3-hydroxy-3-methylglutaryl-coenzyme A lyase deficiency. J Inherit Metab Dis. 1987;10:399.

52. Menao S, López-Viñas E, Mir C, Puisac B, Gratacós E, Arnedo M, et al. Ten novel HMGCL mutations in 24 patients of different origin with 3-hydroxy-3-methyl-glutaric aciduria. Hum Mutat. 2009;30:E520-529.

53. Mir C, Lopez-Viñas E, Aledo R, Puisac B, Rizzo C, Dionisi-Vici C, et al. A single-residue mutation, G203E, causes 3-hydroxy-3-methylglutaric aciduria by occluding the substrate channel in the 3D structural model of HMG-CoA lyase. J Inherit Metab Dis. 2006;29:64–70.

54. Mitchell GA, Jakobs C, Gibson KM, Robert MF, Burlina A, Dionisi-Vici C, et al. Molecular prenatal diagnosis of 3-hydroxy-3-methylglutaryl CoA lyase deficiency. Prenat Diagn. 1995;15:725–9.

55. Mitchell GA, Ozand PT, Robert MF, Ashmarina L, Roberts J, Gibson KM, et al. HMG CoA lyase deficiency: identification of five causal point mutations in codons 41 and 42, including a frequent Saudi Arabian mutation, R41Q. Am J Hum Genet. 1998;62:295–300.

56. Moses SW, Aviram M, Geiger R, Berger R, Smit PC. 3-Hydroxy-3-methylglutaryl-coenzyme A lyase deficiency. J Inherit Metab Dis. 1989;12:341–2.

57. Muñoz-Bonet JI, Ortega-Sánchez MDC, León Guijarro JL. Management and long-term evolution of a patient with 3-hydroxy-3-methylglutaryl-coenzyme A lyase deficiency. Ital J Pediatr. 2017;43:12.

58. Muroi J, Yorifuji T, Uematsu A, Nakahata T. Cerebral infarction and pancreatitis: possible complications of patients with 3-hydroxy-3-methylglutaryl-CoA lyase deficiency. J Inherit Metab Dis. 2000;23:636–7.

59. Muroi J, Yorifuji T, Uematsu A, Shigematsu Y, Onigata K, Maruyama H, et al. Molecular and clinical analysis of Japanese patients with 3-hydroxy-3-methylglutaryl CoA lyase (HL) deficiency. Hum Genet. 2000;107:320–6.

60. Norman EJ, Denton MD, Berry HK. Gas-chromatographic/mass spectrometric detection of 3-hydroxy-3-methylglutaryl-CoA lyase deficiency in double first cousins. Clin Chem. 1982;28:137–40.

61. Ozand PT, al Aqeel A, Gascon G, Brismar J, Thomas E, Gleispach H. 3-Hydroxy-3-methylglutaryl-coenzyme A (HMG-CoA) lyase deficiency in Saudi Arabia. J Inherit Metab Dis. 1991;14:174–88.

62. Pié J, López-Viñas E, Puisac B, Menao S, Pié A, Casale C, et al. Molecular genetics of HMG-CoA lyase deficiency. Mol Genet Metab. 2007;92:198–209.

63. Pierron S, Giudicelli H, Moreigne M, Khalfi A, Touati G, Caruba C, et al. [Late onset 3-HMG-CoA lyase deficiency: a rare but treatable disorder]. Arch Pediatr. 2010;17:10–3.

64. Pipitone A, Raval DB, Duis J, Vernon H, Martin R, Hamosh A, et al. The management of pregnancy and delivery in 3-hydroxy-3-methylglutaryl-CoA lyase deficiency. Am J Med Genet A. 2016;170:1600–2.

65. Plöchl E, Bachmann C, Colombo JP, Gibson KM. [3-hydroxy-3-methylglutaraturia. Clinical aspects, follow-up and therapy in a young child]. Klin Padiatr. 1990;202:76–80.

66. Plöchl E, Colombo JP, Wermuth B, Gibson KM. Increased plasma amylase in the family of a patient with 3-hydroxy-3-methylglutaryl-coenzyme A lyase deficiency. Clin Chem. 1992;38:307–9.

67. Ploechl E, Bachmann C, Colombo JP, Gibson KM. 3-Hydroxy-3-methylglutaryl-CoA-lyase deficiency. J Inherit Metab Dis. 1989;12:343.

68. Pospísilová E, Mrázová L, Hrdá J, Martincová O, Zeman J. Biochemical and molecular analyses in three patients with 3-hydroxy-3-methylglutaric aciduria. J Inherit Metab Dis. 2003;26:433–41.

69. Puisac B, Teresa-Rodrigo ME, Arnedo M, Gil-Rodríguez MC, Pérez-Cerdá C, Ribes A, et al. Analysis of aberrant splicing and nonsense-mediated decay of the stop codon mutations c.109G>T and c.504_505delCT in 7 patients with HMG-CoA lyase deficiency. Mol Genet Metab. 2013;108:232–40.

70. Reimão S, Morgado C, Almeida IT, Silva M, Corte Real H, Campos J. 3-Hydroxy-3-methylglutaryl-coenzyme A lyase deficiency: initial presentation in a young adult. J Inherit Metab Dis. 2009;32 Suppl 1:S49-52.

71. Ribes A, Briones P, Vilaseca MA, Baraibar R, Gairi JM. Sudden death in an infant with 3-hydroxy-3-methylglutaryl-CoA lyase deficiency. J Inherit Metab Dis. 1990;13:752–3.

72. Robinson BH, Oei J, Sherwood WG, Slyper AH, Heininger J, Mamer OA. Hydroxymethylglutaryl CoA lyase deficiency: features resembling Reye syndrome. Neurology. 1980;30 7 Pt 1:714–8.

73. Roe CR, Millington DS, Maltby DA. Identification of 3-methylglutarylcarnitine. A new diagnostic metabolite of 3-hydroxy-3-methylglutaryl-coenzyme A lyase deficiency. J Clin Invest. 1986;77:1391–4.

74. Roland, D, Jissendi, P, Briand, G, Dobbelaere, D. Compared brain and urine MRS spectrum in 5 patients with 3-hydroxy-3-methylglutaryl coenzyme a lyase deficiency. J Inherit Metab Dis. 2010;33 (Suppl 1):54.

75. Roland D, Jissendi-Tchofo P, Briand G, Vamecq J, Fontaine M, Ultré V, et al. Coupled brain and urine spectroscopy - in vivo metabolomic characterization of HMG-CoA lyase deficiency in 5 patients. Mol Genet Metab. 2017;121:111–8.

76. Santarelli F, Cassanello M, Enea A, Poma F, D’Onofrio V, Guala G, et al. A neonatal case of 3-hydroxy-3-methylglutaric-coenzyme A lyase deficiency. Ital J Pediatr. 2013;39:33.

77. Santosa D, Donner MG, Vom Dahl S, Fleisch M, Hoehn T, Mayatepek E, et al. Favourable Outcome in Two Pregnancies in a Patient with 3-Hydroxy-3-Methylglutaryl-CoA Lyase Deficiency. JIMD Rep. 2017;37:1–5.

78. Schiergens KA, Staudigl M, Borggraefe I, Maier EM. Neurological Sequelae due to Inborn Metabolic Diseases in Pediatric Refugees: Challenges in Treating the Untreated. Neuropediatrics. 2018;49:363–8.

79. Schutgens RB, Heymans H, Ketel A, Veder HA, Duran M, Ketting D, et al. Lethal hypoglycemia in a child with a deficiency of 3-hydroxy-3-methylglutarylcoenzyme A lyase. J Pediatr. 1979;94:89–91.

80. Shilkin R, Wilson G, Owles E. 3-Hydroxy-3-methylglutaryl coenzyme A lyase deficiency. Follow-up of first described case. Acta Paediatr Scand. 1981;70:265–8.

81. Sovik O, Sweetman L, Gibson KM, Nyhan WL. Genetic complementation analysis of 3-hydroxy-3-methylglutaryl-coenzyme A lyase deficiency in cultured fibroblasts. Am J Hum Genet. 1984;36:791–801.

82. Stacey TE, de Sousa C, Tracey BM, Whitelaw A, Mistry J, Timbrell P, et al. Dizygotic twins with 3-hydroxy-3-methylglutaric aciduria; unusual presentation, family studies and dietary management. Eur J Pediatr. 1985;144:177–81.

83. Sulaiman RA, Al-Nemer M, Khan R, Almasned M, Handoum BS, Al-Hassnan ZN. Successful Management of Pregnancies in Patients with Inherited Disorders of Ketone Body Metabolism. JIMD Rep. 2018;38:41–4.

84. Thompson GN, Chalmers RA, Halliday D. The contribution of protein catabolism to metabolic decompensation in 3-hydroxy-3-methylglutaric aciduria. Eur J Pediatr. 1990;149:346–50.

85. Tracey, BM, Stacey, TE, Chalmers, RA. Urinary and Plasma Organic Acids in Dizygotic Twin Siblings with 3-Hydroxy-3-methylglutaric Aciduria, Studied by Gas Chromatography and Mass Spectrometry Using Fused Silica Capillary Columns. J Inherit Metab Dis. 1983;6 (Suppl 2):125–6.

86. van der Knaap MS, Bakker HD, Valk J. MR imaging and proton spectroscopy in 3-hydroxy-3-methylglutaryl coenzyme A lyase deficiency. AJNR Am J Neuroradiol. 1998;19:378–82.

87. Vargas CR, Sitta A, Schmitt G, Ferreira GC, Cardoso ML, Coelho D, et al. Incidence of 3-hydroxy-3-methylglutaryl-coenzyme A lyase (HL) deficiency in Brazil, South America. J Inherit Metab Dis. 2008;31 Suppl 3:511–5.

88. Vilarinho L, Cardoso ML, Rabier D, Rolland MO. 3-Hydroxy-3-methylglutaric aciduria in Portuguese population. J Inherit Metab Dis. 1993;16:154–5.

89. Walter JH, Clayton PT, Leonard JV. 3-Hydroxy-3-methylglutaryl-CoA lyase deficiency. J Inherit Metab Dis. 1986;9:287–8.

90. Wanders RJ, Zoeters PH, Schutgens RB, de Klerk JB, Duran M, Wadman SK, et al. Rapid diagnosis of 3-hydroxy-3-methylglutaryl-coenzyme A lyase deficiency via enzyme activity measurements in leukocytes or platelets using a simple spectrophotometric method. Clin Chim Acta. 1990;189:327–34.

91. Wang SP, Robert MF, Gibson KM, Wanders RJ, Mitchell GA. 3-Hydroxy-3-methylglutaryl CoA lyase (HL): mouse and human HL gene (HMGCL) cloning and detection of large gene deletions in two unrelated HL-deficient patients. Genomics. 1996;33:99–104.

92. Wilson WG, Cass MB, Søvik O, Gibson KM, Sweetman L. A child with acute pancreatitis and recurrent hypoglycemia due to 3-hydroxy-3-methylglutaryl-CoA lyase deficiency. Eur J Pediatr. 1984;142:289–91.

93. Wysocki SJ, Wilkinson SP, Hähnel R, Wong CY, Panegyres PK. 3-Hydroxy-3-methylglutaric aciduria, combined with 3-methylglutaconic aciduria. Clin Chim Acta. 1976;70:399–406.

94. Wysocki SJ, Hähnel R. 3-Hydroxy-3-methylglutaric aciduria: deficiency of 3-hydroxy-3-methylglutaryl coenzyme A lyase. Clin Chim Acta. 1976;71:349–51.

95. Wysocki SJ, Hähnel R. 3-Hydroxy-3-methylglutaryl-coenzyme a lyase deficiency: a review. J Inherit Metab Dis. 1986;9:225–33.

96. Wysocki SJ, Hähnel R. 3-Hydroxy-3-methylglutaric aciduria: 3-hydroxy-3-methylglutaryl-coenzyme A lyase levels in leucocytes. Clin Chim Acta. 1976;73:373–5.

97. Wysocki SJ, Hähnel R. 3-Methylcrotonylglycine excretion in 3-hydroxy-3-methylglutaric aciduria. Clin Chim Acta. 1978;86:101–8.

98. Yalçinkaya C, Dinçer A, Gündüz E, Fiçicioğlu C, Koçer N, Aydin A. MRI and MRS in HMG-CoA lyase deficiency. Pediatr Neurol. 1999;20:375–80.

99. Yilmaz O, Kitchen S, Pinto A, Daly A, Gerrard A, Hoban R, et al. [3-hydroxy-3-methylglutaryl-CoA lyase deficiency: a case report and literature review]. Nutr Hosp. 2018;35:237–44.

100. Yoshida I, Søvik O, Sweetman L, Nyhan WL. Metabolism of leucine in fibroblasts from patients with deficiencies in each of the major catabolic enzymes: branched-chain ketoacid dehydrogenase, isovaleryl-CoA dehydrogenase, 3-methylcrotonyl-CoA carboxylase, 3-methylglutaconyl-CoA hydratase, and 3-hydroxy-3-methylglutaryl-CoA lyase. J Neurogenet. 1985;2:413–24.

101. Yýlmaz Y, Ozdemir N, Ekinci G, Baykal T, Kocaman C. Corticospinal tract involvement in a patient with 3-HMG coenzyme A lyase deficiency. Pediatr Neurol. 2006;35:139–41.

102. Zafeiriou DI, Vargiami E, Mayapetek E, Augoustidou-Savvopoulou P, Mitchell GA. 3-Hydroxy-3-methylglutaryl coenzyme a lyase deficiency with reversible white matter changes after treatment. Pediatr Neurol. 2007;37:47–50.

103. Zapater N, Pié J, Lloberas J, Rolland MO, Leroux B, Vidailhet M, et al. Two missense point mutations in different alleles in the 3-hydroxy-3-methylglutaryl coenzyme A lyase gene produce 3-hydroxy-3-methylglutaric aciduria in a French patient. Arch Biochem Biophys. 1998;358:197–203.

104. Zoghbi HY, Spence JE, Beaudet AL, O’Brien WE, Goodman CJ, Gibson KM. Atypical presentation and neuropathological studies in 3-hydroxy-3-methylglutaryl-CoA lyase deficiency. Ann Neurol. 1986;20:367–9.
