# Supplementary material for: 3-hydroxy-3-methylglutaryl-coenzyme A lyase deficiency: one disease - many faces
Source: Orphanet J Rare Dis. 2020 Feb 14;15:48. doi: 10.1186/s13023-020-1319-7 (PMC7023732; doi:10.1186/s13023-020-1319-7)
Supplement: Supplementary file 2 — Additional file 2: Table S2. Publications that were additionally included for the overview on all known mutations in HMGCL. [file 13023_2020_1319_MOESM2_ESM.docx]

**Table S2: Publications that were additionally included for the overview on all known mutations in *HMGCL***

1. Al-Sayed M, Imtiaz F, Alsmadi OA, Rashed MS, Meyer BF. Mutations underlying 3-hydroxy-3-methylglutaryl CoA lyase deficiency in the Saudi population. BMC Med Genet. 2006;7:86.

2. Mitchell GA, Robert MF, Hruz PW, Wang S, Fontaine G, Behnke CE, et al. 3-Hydroxy-3-methylglutaryl coenzyme A lyase (HL). Cloning of human and chicken liver HL cDNAs and characterization of a mutation causing human HL deficiency. J Biol Chem. 1993;268:4376–81.

3. Pié J, Casals N, Casale CH, Buesa C, Mascaró C, Barceló A, et al. A nonsense mutation in the 3-hydroxy-3-methylglutaryl-CoA lyase gene produces exon skipping in two patients of different origin with 3-hydroxy-3-methylglutaryl-CoA lyase deficiency. Biochem J. 1997;323 ( Pt 2):329–35.

4. Puisac B, Ramos M, Arnedo M, Menao S, Gil-Rodríguez MC, Teresa-Rodrigo ME, et al. Characterization of splice variants of the genes encoding human mitochondrial HMG-CoA lyase and HMG-CoA synthase, the main enzymes of the ketogenesis pathway. Mol Biol Rep. 2012;39:4777–85.

5. Roberts JR, Mitchell GA, Miziorko HM. Modeling of a mutation responsible for human 3-hydroxy-3-methylglutaryl-CoA lyase deficiency implicates histidine 233 as an active site residue. J Biol Chem. 1996;271:24604–9.
